# Zinc Phthalocyanine Core‐First Star Polymers Through Nitroxide Mediated Polymerization and Nitroxide Exchange Reaction

**DOI:** 10.1002/marc.202400601

**Published:** 2024-09-28

**Authors:** Erem Ahmetali, Azra Kocaarslan, Stefan Bräse, Patrick Théato, M. Kasım Şener

**Affiliations:** ^1^ Institute of Organic Chemistry (IOC) Karlsruhe Institute of Technology (KIT) Kaiserstraße 12 76131 Karlsruhe Germany; ^2^ Department of Chemistry Yıldız Technical University Istanbul 34210 Turkey; ^3^ Institute for Chemical Technology and Polymer Chemistry Karlsruhe Institute of Technology (KIT) Engesserstraße 18 76131 Karlsruhe Germany; ^4^ Institute of Biological and Chemical Systems – Functional Molecular Systems (IBCS‐FMS) Karlsruhe Institute of Technology Hermann‐von‐Helmholtz‐Platz 1 76344 Eggenstein‐Leopoldshafen Germany; ^5^ Soft Matter Synthesis Laboratory – Institute for Biological Interfaces III (IBG‐3) Karlsruhe Institute of Technology (KIT) Hermann‐von‐Helmholtz‐Platz 1 76344 Eggenstein‐Leopoldshafen Germany

**Keywords:** core‐first star polymer, nitroxide exchange reaction, nitroxide mediated polymerization, phthalocyanine, polymeric phthalocyanine, star polymer

## Abstract

Nitroxide‐mediated polymerization (NMP) and nitroxide exchange reaction (NER) are very efficient methodologies that require only suitable alkoxyamine derivatives and create different polymeric architectures in a controlled manner. Herein, the synthesis of star polymers containing TEMPO‐substituted symmetric zinc phthalocyanine (ZnPc) is presented via NMP and NER. Moreover, linear polymer formation is conducted in a single arm on TEMPO‐substituted asymmetric ZnPc to elucidate the properties of star polymers. All linear and star polymers are characterized by FT‐IR, UV–vis, fluorescence, GPC, NMR, and EPR techniques. The results show that the proposed reactions are capable of forming controlled star‐shaped polymers. The increasing arm number (from a single to four arms) results in variable dispersity values (*Đ*) (1.2–3) due to different arm lengths, especially in NMP. However, this difficulty has been overcome via NER, and star polymers have been successfully synthesized with relatively low molecular weight (30 K > 10 K) and low dispersity (1.2–1.9). The results clearly indicate that while styrene and 4‐vinyl benzyl chloride monomers are introduced to the structure equally, star polymers with phthalocyanine can be synthesized in a controlled manner, and their quarternized derivatives have the potential to be effective as photoactive agents in photodynamic therapy.

## Graphical abstract

The synthesis and characterization of zinc phthalocyanine core star polymers and zinc phthalocyanine end‐functionalized linear polymers are successfully achieved via using of styrene and vinyl benzyl chloride monomers. The reactions are carried out with suitable radical and alkoxyamine initiators via nitroxide‐mediated polymerization (NMP) and nitroxide exchange reaction (NER). The results show that selecting an equal amount of monomers together with the initiator, which is needed for NER, can provide a more controlled polymer design with a post‐polymerization modification (PPM) opportunity.

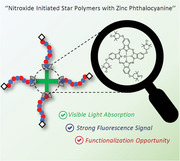

## 1 Introduction

The advancement of reversible‐deactivation radical polymerization (RDRP) has significantly influenced the field of polymer research by enabling the design of various compositions, topologies, and functionalities under comparatively mild polymerization conditions.^[^ ^1^ ^]^ Atom transfer radical polymerization (ATRP),^[^ ^2^ ^]^ reversible addition‐fragmentation chain transfer (RAFT)^[^ ^3^ ^]^ polymerization, and nitroxide‐mediated polymerization (NMP) are among the main types of RDRP methods for the preparation of well‐defined polymer architecture. NMP, especially, has always been considered a simple one since, in most cases, it requires only a suitable alkoxyamine for the polymerization system.^[^ ^4^ ^]^ It is based on a thermodynamic equilibrium between dormant macro‐alkoxyamines capped by a nitroxide moiety and actively propagating macroradicals based on a reversible termination mechanism.^[^ ^5^ ^]^ Thus, this method enables the preparation of different polymers with defined molecular weights and low dispersities (*Đ*).^[^ ^6^ ^]^ Furthermore, adding a functional group to an alkoxyamine derivative is highly efficient for creating macromolecules with functional end groups.^[^ ^5^, ^7^ ^]^ NMP also allows radical exchange reactions between the nitroxides of different alkoxyamine derivatives. In this process, thermal C─O bond homolysis of alkoxyamines leads to two types of radicals: a transient carbon‐centered radical and a persistent nitroxide radical. When the homolysis of an alkoxyamine is performed in the presence of additional nitroxide radicals, the thermodynamically favored mixed derivatives are obtained.^[^ ^8^, ^9^ ^]^ This process, named nitroxide exchange reaction (NER), has been successfully explored for preparing dynamic polymers and networks.^[^ ^10^, ^11^, ^12^, ^13^, ^14^, ^15^ ^]^

NMP and NER have also been effective in preparing star polymers.^[^ ^16^, ^17^, ^18^, ^19^ ^]^ They have received significant attention due to their unique properties, arising from the highly branched 3D structure like low viscosity and high flexibility. Even though star polymers were first described in 1948 by Schaefgen and Flory, the synthesis of well‐defined multiarmed polymers posed a challenge until living polymerization techniques emerged.^[^ ^20^ ^]^ However, they have revealed their potential in many biomedical applications, including drug/gene delivery, diagnosis, and antibacterial/antifouling coatings.^[^ ^21^ ^]^ Star polymers are a type of macromolecule characterized by multiple linear chains, or arms, radiating from a central branching point. Two fundamental strategies are frequently used to synthesize star polymers: the arm‐first and core‐first methods.^[^ ^22^ ^]^ In the arm‐first method, a well‐defined preformed linear arm is attached to the core molecule via covalent interactions. This strategy precisely controls the length of arms on the macromolecular structure. On the other hand, the core‐first method grows polymeric arms directly from a multifunctional initiator. Even though the core‐first method necessitates meticulous effort for the design and synthesis of an appropriate multifunctional initiator (core), it is a chance to create pure star polymers with a specific number of arms that can be isolated from the crude reaction (such as unreacted monomers, ligands, catalyst and so on).^[^ ^20^ ^]^ Up to now, many different organic compounds,^[^ ^23^ ^]^ organic–inorganic hybrid materials,^[^ ^24^ ^]^ and macrocyclic structures^[^ ^25^ ^]^ have been successfully utilized as a core in the synthesis of star polymers.

Phthalocyanines (Pcs) are one class of macrocyclic compounds consisting of four isoindoline units and fulfill the definition of the multifunctional core to a high degree.^[^ ^26^ ^]^ It is possible to synthesize four‐armed or eight‐armed Pcs by changing the starting materials (i.e., 4‐nitrophthalonitrile, 4,5‐dichlorophthalonitrile). Also, they can form metal complexes with different ions, such as zinc and cobalt, thanks to the nitrogen atoms at their meso positions.^[^ ^27^, ^28^ ^]^ Moreover, Pcs have eighteen π‐electron conjugated ring systems, giving them high electron transfer abilities, thermal‐chemical stability, absorption capability in the visible region, and fluorescence behavior.^[^ ^29^, ^30^, ^31^ ^]^ Incorporation of Pcs into polymeric materials is an efficient way to enrich polymer structures with these features.^[^ ^32^, ^33^ ^]^ Until now, although the synthesis of different star polymers with Pcs has been successfully achieved by ATRP,^[^ ^34^, ^35^ ^]^ RAFT,^[^ ^36^ ^]^ and ring‐opening polymerization (ROP),^[^ ^37^ ^]^ no example has been carried out with NMP and NER.

In this study, four‐armed star polymers (**SP**) with zinc phthalocyanine (ZnPc) consisting of various polymer compositions were prepared by NMP and NER. Benzoyl peroxide (**I1**) and 2,2,6,6‐tetramethyl‐1‐(1‐phenyl ethoxy)piperidine (**I2**) were selected as initiators for NMP and NER, respectively. While star polymers were prepared from styrene (St) and 4‐vinyl benzyl chloride (VBCl) monomers, four‐armed TEMPO substituted ZnPc was used as a multifunctional core to bring the polymer chains together on the macrocyclic structure. Additionally, the ZnPc end‐functionalized linear polymers (**LP**) were synthesized using one‐armed TEMPO substituted ZnPc. This attempt aims to explain better the different findings that could result from multi‐arm star polymer formation. To our knowledge, this paper will be the first investigation to produce core‐first star polymer formation through NMP and NER, including the phthalocyanine structure.

## 2 Results and Discussion

**Figure 1:**
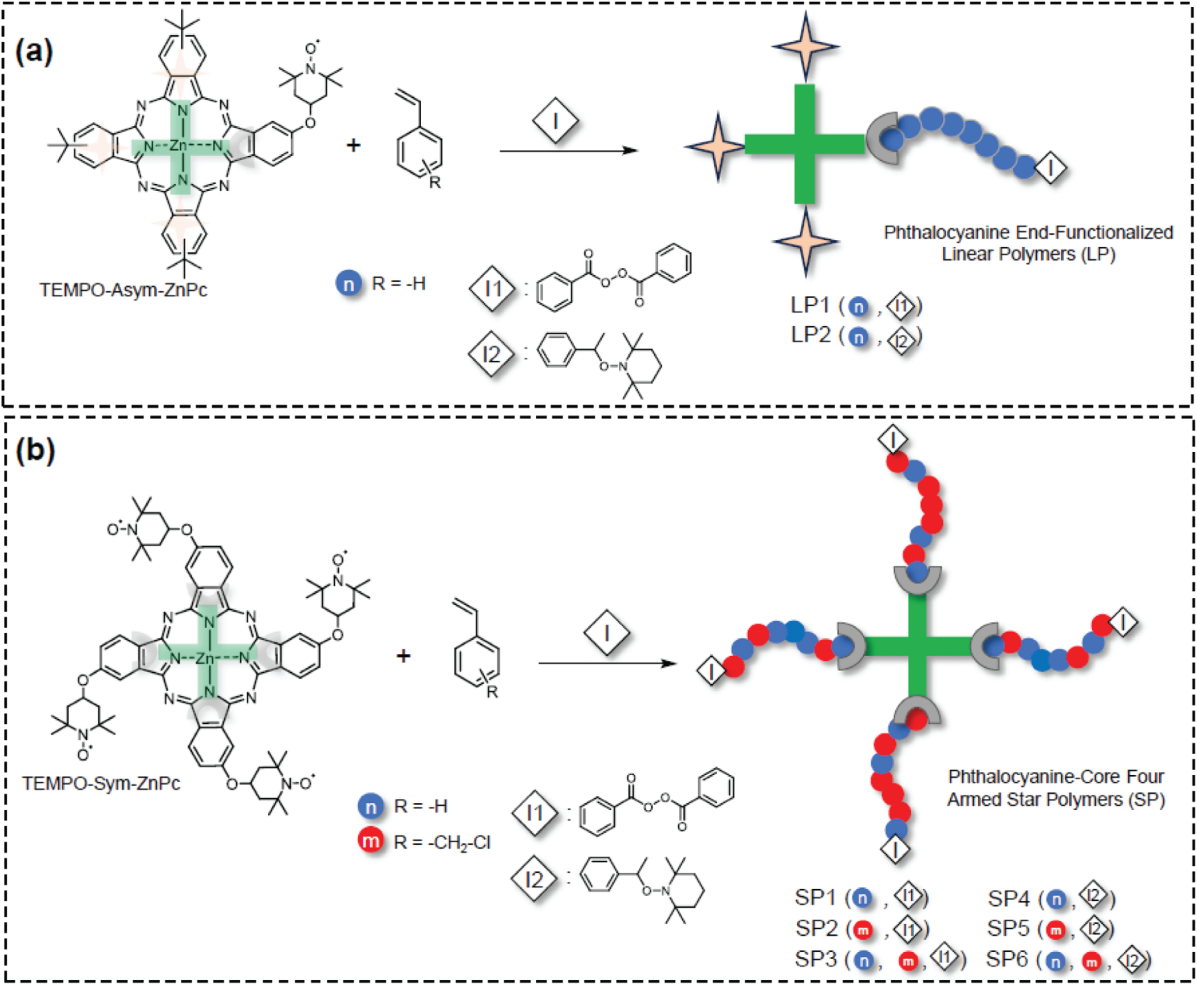
The schematic representation of linear and star‐shaped polymers via nitroxide‐mediated polymerization and nitroxide exchange reaction. a) Synthesis of linear polystyrene with the TEMPO‐Asym‐ZnPc end functionality (**LP1**, **LP2**) in the presence of benzyl radical and TEMPO initiator. b) Synthesis of star‐shaped homo‐ and random block copolymers with TEMPO‐Sym‐ZnPc (**SP1**‐**SP6**) in the presence of benzyl radical and TEMPO initiator.

The current study focuses on linear and star‐shaped polymer formation through TEMPO‐decorated zinc phthalocyanine (ZnPc) via nitroxide‐mediated polymerization (NMP) as well as nitroxide exchange reaction (NER). For this purpose, previously reported TEMPO‐Sym‐ZnPc^[^ ^38^ ^]^ (**Figure** **1**b) has been adopted together with the novel compound TEMPO‐Asym‐ZnPc (Figure 1a) in nitroxide‐based polymerization. The synthesis of TEMPO‐Asym‐ZnPc was achieved by a reaction of 4‐*tert*‐butylphthalonitrile and TEMPO‐phthalonitrile in the presence of n‐hexanol, 1,8‐Diazabicyclo[5.4.0]undec‐7‐ene (DBU) and Zn(CH_3_COO)_2_ at 160 °C (Supporting Information).

The FT‐IR spectrum of TEMPO‐Asym‐ZnPc shows the stretching vibrations belonging to aromatic C─H (3071 cm^−1^), aliphatic C─H (2952 cm^−1^), and radical N─O (1373 cm^−1^) bonds of Pc (Figure S1, Supporting Information). In the MALDI‐TOF mass spectrum, [M]^+^ found an *m/z* of 914.74 in harmony with the expected value (914.38) (Figure S2, Supporting Information). The EPR spectrum proved the presence of radicals belonging to the TEMPO units in the structure, similar to the bare TEMPO radical (Figure S3, Supporting Information).

**Figure 2:**
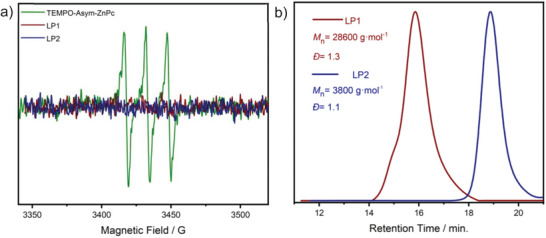
a) The EPR spectra of TEMPO‐Asym‐ZnPc (green), **LP1** (red), and **LP2** (blue) b) THF‐SEC traces of **LP1** (red) and **LP2** (blue) and normalized RI detector response.

Accordingly, linear polymers were prepared via NMP and NER (Figure 1a). Nitroxide‐stabilized free radicals have been previously demonstrated to facilitate the breakdown of peroxide initiators, potentially aiding in the simultaneous initiation of all polymeric chains.^[^ ^39^ ^]^ In this regard, to synthesize **LP1**, TEMPO‐Asym‐ZnPc was reacted with styrene (St) and benzoyl peroxide (**I1**) in toluene at 130 °C for 24 h using nitroxide‐mediated polymerization (NMP). Subsequently, polymerization was performed using initiator that generates free radicals via nitroxide exchange reaction (NER). This process involves heating the polymer solution with an alkoxyamine‐capped compound in the presence of a conjugation partner containing a free nitroxide moiety. Similar to NMP, the carbon–oxygen bond in the alkoxyamine is thermally cleaved, regenerating the propagating radical and releasing a free nitroxide radical.^[^ ^40^ ^]^ Therefore, 2,2,6,6‐tetramethyl‐1‐(1‐phenyl ethoxy)piperidine (**I2**) was used as the initial precursor instead of **I1** to synthesize **LP2**. While the EPR spectra of TEMPO‐Asym‐ZnPc confirmed the presence of radicals linked to TEMPO units within the structure before polymerization, conversely, after the polymerization, these radical bands diminished, indicating consumption of TEMPO‐Asym‐ZnPc and transformation to **LP1** and **LP2** (**Figure** **2**a). The molecular weight characteristics of the linear polymers are summarized in Figure 2b. SEC traces of **LP1** and **LP2** showed that NER provided slightly better control over the polymerization with good dispersities (*Ð* = 1.3 and 1.1, respectively). The FT‐IR spectra of linear polymers exhibited the aliphatic bands of Pc at 2916 cm^−1^ next to the aromatic bands of polystyrene at 3024 cm^−1^ (Figure S5, Supporting Information). UV–vis spectroscopy is an important technique to prove the presence of phthalocyanine structure in the compounds. They have three characteristic absorption bands: i) the Q band found at 650–750 nm in the visible region, ii) the B (Soret) band observed at 300–350 nm in the ultraviolet region, and iii) The Q vibrational (Q_vib_) band at 610–615 nm in the bluer region than the Q band. The UV–vis spectra exhibited these characteristic Pc bands at 350, 610, and 676 nm, respectively, for both **LP1** and **LP2** (Figure S6, Supporting Information). Further characterization was completed by ^1^H‐NMR and ^13^C‐NMR spectroscopy (Figures S7 and S8, Supporting Information).

The successful synthesis of star‐shaped core‐first ZnPc polymer was accomplished by the NMP in the presence of **I1** (Figure 1b). **SP1, SP2, and SP3** were obtained in the same manner as in **LP1,** and the reaction was carried out in toluene in the presence of **I1**. In addition to the homopolymerization of St, the homopolymerization of vinyl benzyl chloride (VBCl) and the copolymerization of St and VBCl were conducted. The data is summarized in **Table** **1**.

**Table 1:** Molecular weight characteristics of the star‐shaped polymers synthesized via NMP.

Polymer	Monomer	Conv. %^a)^	*M*_n, SEC‐RI_ ^b)^ g mol^−1^	*M*_n, SEC‐LS_ ^c)^ g mol^−1^	*M*_n, NMR_ ^d)^ g mol^−1^	*M*_n, calc._ ^e)^ g mol^−1^	*Ð*
**SP1**	St	68	32 940	35 370	38 130	85 500	1.7
**SP2**	VBCl	54	44 770	50 800	32 250	34 060	3.0
**SP3**	St: VBCl	79	36 360	31 170	57 070	33 000	2.7

TEMPO‐Sym‐ZnPc: I1 = 1:1.5. Reaction time = 24 h.

^a)^ Conversion was calculated gravimetrically.

^b)^ Determined by SEC with refractive index detector.

^c)^ Determined by SEC with light scattering detector.

^d)^ Determined by ^1^H‐NMR spectroscopy.

^e)^ $Mn=\frac{\left[monomer\right]}{\left[TEMPO-Sym-ZnPc\right]}\times M_{w,monomer}\times conversion+M_{w,TEMPO-Sym-ZnPc}$

**Figure 3:**
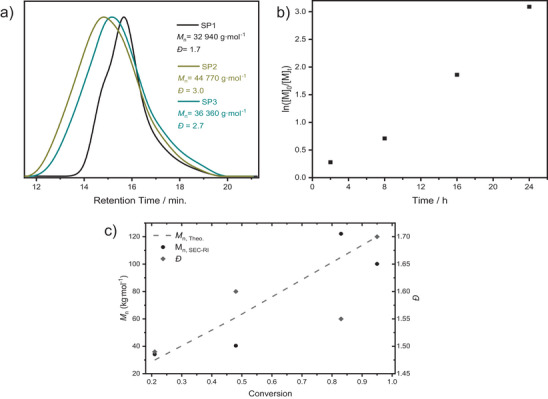
a) THF‐SEC elution curves of star polymers (**SP1**–**SP3**) initiated with **I1**, b) ln/[M]_0_/[M]_t_) versus time plots for star‐shaped styrene polymerization initiated with **I1**, c) *M*_n_ versus conversion (■) and *Đ* versus conversion plots for star‐shaped styrene polymerization initiated with **I1**. Theoretical *M*_n_ values are calculated with the respective equations (Table 1 footnote e).

The observed variance in *M*_n_ can be explained by considering the smaller hydrodynamic volumes of star polymers compared to their linear analogous equivalent molecular weights.^[^ ^41^ ^]^ In this regard, the *M*_n_ values were calculated by ^1^H‐NMR spectroscopy of the star polymers (**SP1–SP3**). The appearance of the characteristic aromatic protons (6.2–7.2 ppm) and main chain protons (1.1–2.2 ppm) of PS confirmed the successful polymerization. Additionally, a triplet band of the H_2_C‐O in the TEMPO unit connected to the St was observed at 3.67 ppm (Figures S9 and S10, Supporting Information). Based on that, the star polymer *M*_n_ values were determined by the integration ratio of aromatic protons to respective protons, which belong to the TEMPO unit. The determined *M*_n_ values by the ^1^H‐NMR were in excellent agreement with the calculated values (Table 1). The dispersity *(Ð)* determined with SEC at 20% and 40% conversions are 1.45 and 1.55, respectively, indicating that the polymerization was slightly more controlled at low conversion. Moreover, *Ð* reached 1.70 at 90% conversion. This means that the effects of side reactions can no longer be ignored at high conversion (**Figure** **3**).

On the other hand, the concept was tested by using **I2** instead of **I1** while keeping all other components the same to investigate the effect of NER on polymer synthesis (Figure 1b). The synthesis of **SP4** showed that during the polymerization of St, the *Ð* of the resulting polymers decreased as the concentration of the alkoxyamine increased.^[^ ^42^ ^]^ This indicates that higher alkoxyamine concentrations help suppress side reactions that would otherwise interfere with the controlled polymerization process. The polymerization results are summarized in **Table** **2**.

**Table 2:** Molecular weight characteristics of the star‐shaped polymers synthesized by NMP NER.

Polymer	Monomer	Time (day)	Conv. %^a)^	*M*_n, SEC‐RI_ ^b)^ g mol^−1^	*M*_n, SEC‐LS_ ^c)^ g mol^−1^	*M*_n, NMR_ ^d)^ g mol^−1^	*Ð*
**SP4**	St	7	74	5198	6397	33 550	1.2
**SP5**	VBCl	3	98	13 986	16 500	33 000	1.9
**SP6**	St: VBCl	3	40	8305	10 090	45 330	1.4

TEMPO‐Sym‐ZnPc: I2 = 1:4. Reaction time = 24 h;

^a)^ Conversion was calculated gravimetrically;

^b)^ Determined by SEC with refractive index detector;

^c)^ Determined by SEC with light scattering detector;

^d)^ Determined by ^1^H‐NMR spectroscopy.

**Figure 4:**
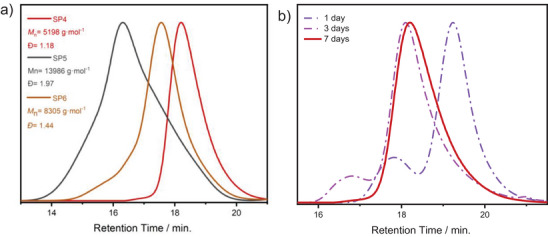
a) THF‐SEC curves of **SP4**–**SP6** via NER. b) Screening of THF‐SEC traces of star‐shaped polymer by time.

Furthermore, the star‐shaped polymer formation via the NER mechanism was followed over time, and molecular weight characteristics are given in **Figure** **4**. Although SEC elution curves of **SP4–SP6** showed a uniform band (Figure 4a), star polymers containing VBCl monomer showed a very broad band that could be attained with different chain lengths in each arm. However, achieving control over the formation of star‐shaped polystyrene homopolymer required a longer reaction time (Figure 4b, red curve). The extension of reaction time from one to seven days caused the disappearance of the second peak, which belongs to the linear polymer with low molecular weight. The presence of the second peak can be attributed to homopolymer formation, proven via the blank experiment. It can be concluded that NER has provided more control over star polymer formation (**SP4–SP6**) at lower molecular weights (*M*_n_ = 10–17 kg·mol^−1^) with lower dispersity (*Đ* = 1.2‐1.9) than NMP does.

**Figure 5:**
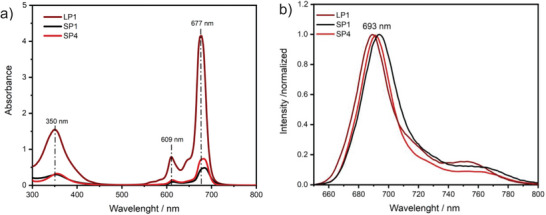
a) UV–vis spectra of **LP1** (claret red), **SP1**(black), and **SP4** (red) in DMF (1 mg mL^−1^, 298 K). **b)** Fluorescence emission spectra of the **LP1** (claret red), **SP1** (black) and **SP4** (red) in DMF (1 mg mL^−1^, 298 K, excitation wavelength  =  612 nm).

Along with the SEC measurements, spectroscopic analysis via FT‐IR, UV–vis, and fluorescence revealed the compositions of resulting star polymers (**SP1**–**SP6**). EPR analysis conclusively demonstrated the absence of residual TEMPO‐ZnPc radicals after polymerization (Figures S13 and S14, Supporting Information). All star‐shaped polymers exhibited strong Pc absorption (≈686 nm) and emission values (≈693 nm) in the visible region (Figures S15–S18, Supporting Information). Results showed that polymers have gained visible light absorption and fluorescence behavior. However, linear polymers have relatively different absorption values in the same concentration (**Figure** **5**a). While the difference between the first series and second series (e.g., between **SP1** and **SP4**) can be explained by the different polymer chain lengths in the arms, the superiority of linear polymers (e.g., **LP1**) is derived from different substituent (*tert*‐butyl groups) in the asymmetric phthalocyanine, which can change the molar absorption coefficient of compounds. In the FT‐IR spectra of compounds, intensities at C─H (1258 cm^−1^) and C─Cl (817 cm^−1^) provided us with an understanding of the monomer content of star polymers. While **SP2** and **SP5** only contained VBCl monomer, the expected molar ratio is 1 in **SP3** and **SP6** (Supporting Information page 10 for all values). In both the **SP1–SP3** and **SP4–SP6** series, the intensity of bands increased based on VBCl content, and these results are in harmony with the expectations related to the molar ratio (Figures S19 and S20, Supporting Information).

**Figure 6:**
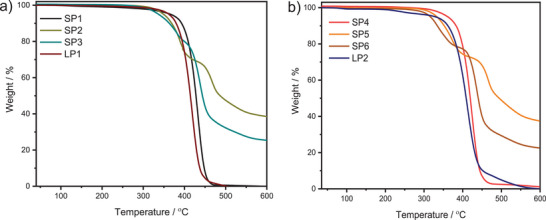
Thermogravimetric analysis (TGA) of a) **SP1** (black), **SP2** (olive), **SP3** (green), and **LP1** (claret red), b) **SP4** (red), **SP5** (orange), **SP6** (brown), and **LP2** (navy blue) with a heating rate of 10 K min^−1^ under a nitrogen flow.

The thermal stability of different star‐shaped homopolymers (**SP1** and **SP2**, **SP4** and **SP5**) and respective copolymers (**SP3** and **SP6**) structures was evaluated by thermogravimetric analysis (TGA) in the temperature range of 40–600 °C, as shown in **Figure** **6**. Star‐shaped **SP1**, **SP4** and **LP1**, **LP2** containing only St monomer indicated a single degradation peak attributed to the primary chain decomposition at 360 °C in agreement with the literature.^[^ ^43^, ^44^ ^]^ Nevertheless, the poly(VBCl) (**SP2** and **SP5**) and poly(St‐*co*‐VBCl) (**SP3** and **SP6**) exhibited two decomposition peaks derived from the VBCl monomer. It starts to degrade from ≈350 °C, which comes from the degradation of ‐CH_2_Cl groups, and then main chain decomposition occurs above ≈450 °C.^[^ ^45^ ^]^ Furthermore, char yields were calculated, which define the remaining mass after degradation. It is clear that star polymers containing VBCl have higher durability, and more substances remained stable after the procedure at 600 °C (36.1% for **SP2** and 34.5% for **SP5**) (**Table** **3**).

**Table 3:** Thermal properties of the linear and star‐shaped polymers.

Polymers	T_5%_ [°C]	T_10%_ [°C]	Char yield @600 °C [%]
**LP1**	361.0	378.6	0.01
**LP2**	329.0	361.8	0.01
**SP1**	358.3	385.0	0.01
**SP2**	336.3	357.3	36.1
**SP3**	326.0	348.1	22.0
**SP4**	360.6	382.2	1.13
**SP5**	321.2	249.3	34.5
**SP6**	310.1	333.4	17.4

T_5%:_ The temperature for which the weight loss is 5% by mass.

T_10%_: The temperature for which the weight loss is 10% by mass.

**Figure 7:**
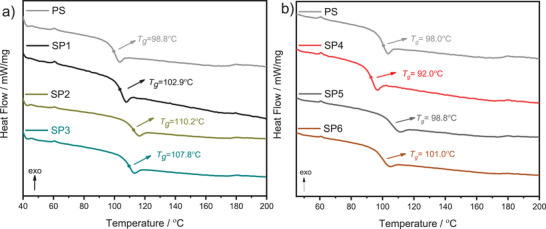
DSC curves of (second heating run) a) star‐shaped polymers **SP1** (black), **SP2** (yellow), and **SP3** (dark cyan) b) star‐shaped polymers **SP4** (red), **SP5** (grey), and **SP6** (orange) from 30 to 200 °C with a heating rate of 10 K min^−1^ under nitrogen flow.

Complementary to TGA, the differential scanning calorimetry (DSC) analysis was carried out on star polymers. The glass transition temperature (*T*_g_) curves and values between 102.0 and 107.8 °C confirmed polystyrene's amorphous behavior. (**Figure** **7**). *T*_g_ depends strongly on the molecular weight, especially lower molecular weights, due to the free volume around the chain ends. Thus, low molecular mass gives lower values of *T*_g_.^[^ ^46^ ^]^ This expected correlation showed itself in our results. **SP1**–**SP3** (polymers derived from **I1**) featured a higher *T*_g_ than **SP4**–**SP6** (polymers derived from **I2**) (average 30.000 g mol^−1^ > 10.000 g mol^−1^).

## 3 Conclusion

In this study, we describe a core‐first star polymer synthesis with controlled molecular composition via nitroxide‐mediated polymerization (NMP) (**SP1–3**) and nitroxide exchange reaction (NER) (**SP4–6**). Symmetric zinc phthalocyanine (ZnPc) was used as a multi‐arm core, and polymeric chains could successfully grow directly on its arms via NMP. However, additional alkoxide radicals could provide more control over the polymerization via NER with ZnPc. The results proved that the molecular weight and dispersity values are dramatically affected by the initiation mechanism which results from the different initiators (benzoyl peroxide (**I1**) and 2,2,6,6‐tetramethyl‐1‐(1‐phenyl ethoxy)piperidine (**I2**). Meanwhile, styrene (St) and vinyl benzyl chloride (VBCl) were used as monomers in the polymerization. Increasing the amount of VBCl gave rise to enhancing the dispersity values. It is thought that this is due to the relatively low reactivity of VBCl compared to St. NMP and NER methodologies, which offer a promising way to produce different types of macromolecular structures like graft, block, and star copolymers using carefully chosen functional materials. It is especially possible to obtain star polymers with a higher photosensitizer (ZnPc) signal by taking advantage of the NER mechanism. Moreover, the presence of chlorine groups on the polymer chain provides the possibility of post‐polymerization modification for further applications. In that sense, the quaternization of star polymers is planned, and they are seen as a promising candidate for photodynamic therapy due to the formation of positive charges.

## Conflict of Interest

The authors declare no conflict of interest.

## Author Contributions

E.A. and A.K. contributed equally to this work. They led the study, performed the experiments, and composed the manuscript. The manuscript was written with A.K., E.A., S.B., P.T., and M.K.Ş. contributions. This paper is dedicated to the late Prof. Dr. Yusuf Yagci, who has been an exceptionally inspiring and supportive mentor for many young polymer chemists.

## Supporting information



Supporting Information

## Data Availability

The data that support the findings of this study are available in the supplementary material of this article.
