# Supplementary material for: Zinc Phthalocyanine Core‐First Star Polymers Through Nitroxide Mediated Polymerization and Nitroxide Exchange Reaction
Source: Macromol Rapid Commun. 2024 Sep 28;46(1):2400601. doi: 10.1002/marc.202400601 (PMC11713867; doi:10.1002/marc.202400601)
Supplement: Supplementary file 1 — Supporting Information [file MARC-46-2400601-s001.docx]

**Supporting Information**

Zinc Phthalocyanine Core‐First Star Polymers Through Nitroxide Mediated Polymerization and Nitroxide Exchange Reaction

Erem Ahmetali*, Azra Kocaarslan*, Stefan Bräse, Patrick Théato and M. Kasım Şener*

**Contents**

[1. Materials 2](#_Toc166084844)

[2. Instrumentation 2](#_Toc166084845)

[3. Synthesis of TEMPO Phthalonitrile (TEMPO-Pht) 3](#_Toc166084846)

[4. Synthesis of TEMPO Symmetrical Zinc Phthalocyanine (TEMPO-Sym-ZnPc) 3](#_Toc166084847)

[5. Synthesis and Characterization of TEMPO Asymmetrical Zinc Phthalocyanine (TEMPO-Asym-ZnPc) 4](#_Toc166084848)

[6. Synthesis of Linear Polymer via I1 Initiator 7](#_Toc166084849)

[7. Synthesis of Linear Polymer via I2 Initator 7](#_Toc166084850)

[8. Synthesis of Star-shaped Polymer via I1 Initiator 8](#_Toc166084851)

[9. Synthesis of Star-shaped Polymer via I2 Initiator 9](#_Toc166084852)

[10. Characterization of Polymers 10](#_Toc166084853)

[References 18](#_Toc166084855)

# 1. Materials

Styrene (Thermo Scientific, 99%) and 4-vinylbenzyl chloride (Merck, 90%) were passed through the column and kept cold before use. Benzoyl peroxide (BPO, Merck Darmstadt, Germany) (**I1**) crystallized from ethanol before use. (1-Bromoethyl)benzene (Alfa Easer, 97%), 2,2,6,6-tetramethyl-1-piperidinyloxy (TEMPO, BLD Pharm), copper powder (Honeywell), N,N,N^I^,N^II^,N^II^-pentamethyldiethylenetriamine (PMDETA, Across, 99%), 4-nitrophthalonitrile (BLD Pharm), 4-*tert*-butylphthalonitrile (BLD Pharm), 4-hydroxy-2,2,6,6-tetramethylpiperidinyloxyl (TEMPOL, BLD Pharm), K_2_CO_3_ (Sigma), acetonitrile (VWR), toluene (Sigma, dehydrated), dimethylformamide (DMF, VWR), dichloromethane (DCM, VWR), methanol (VWR) and tetrahydrofuran (THF, Sigma, dehydrated) were used as received.

# 2. Instrumentation

*General Instruments:* FT-IR spectra of all polymers were recorded with a spectrometer (Bruker). Measurements were performed in a transmittance mode using a 4000 to 400 cm^-1^ spectral range. Mass spectra were measured on a Bruker Microflex LT MALDI TOF-MS. The molecular weight characteristics of all polymers were measured in THF by using size-exclusion chromatography (SEC) *Agilent Technologies 1260 Infinity II* system with a 5 µm *PSS SDV Lux 1000 Å* column (8 x 300 mm) and a 5 µm *PSS SDV Lux 100.000 Å* column (8 x 300 mm). The measurements were performed at 35 °C. The calibration was carried out using different linear polystyrene (PS) standards ranging from 370 to 2.5 x 10^6^ g·mol^-1^. The polymer samples were dissolved at 2 mg·mL^-1^ in THF and filtered over a 0.2 µL filter before measurement. All UV-vis spectra were recorded on a Varian Cary 300 Bio UV-vis spectroscopy with an electronic temperature control cell positioner. Samples were prepared in solvent dimethylformamide (DMF) and measured in a 10 mm quartz cuvette at ambient temperature. Electron Paramagnetic Resonance (EPR) spectroscopy was recorded with BRUKER EMX nano. H-(400 MHz) NMR spectra were recorded on a Bruker AVANCE III Microbay spectrometer using deuterated solvents, calibrated using residual undeuterated solvent or tetramethylsilane as the internal standard. Thermal gravimetric analysis (TGA) was carried out using a TGA 5500 (TA Instruments) at a heating rate of 10 K/ min^−1^ under a nitrogen atmosphere up to 800 °C. Differential scanning calorimetry (DSC) was conducted using a DSC Q200 (TA Instruments) ranging from 30 °C to 200 °C with a 10 K/min^−1^ scan rate.

# 3. Synthesis of TEMPO Phthalonitrile (TEMPO-Pht)

TEMPO Phthalonitrile (TEMPO-Pht) was synthesized according to a modified literature procedure.^[1]^ NO_2_-Pht (1.0 equiv) and TEMPOL (1.35 equiv) were dissolved in DMF, and K_2_CO_3_ (2.6 equiv) was added slowly. It was stirred for 72 hours at room temperature (The reaction was followed by TLC in DCM). After that, the reaction mixture was poured into an ice-water mixture, and the precipitated substance was filtered and dried in a vacuum oven at 40 °C. The residue was dissolved in chloroform and filtrating. After evaporating, a pure solid substance was obtained.

# 4. Synthesis of TEMPO Symmetrical Zinc Phthalocyanine (TEMPO-Sym-ZnPc)

Symmetrical TEMPO substituted zinc phthalocyanine (TEMPO-Sym-ZnPc) was synthesized according to a modified literature procedure.^[1]^ To the suspension of TEMPO-Pht (1.0 equiv) in n-hexanol, Zn(CH_3_COO)_2_ (0.25 equiv) and a few drops of DBU were added. It was stirred for 5 h at 130 ^o^C. After that, the reaction mixture was poured into n-hexane, and the precipitate was centrifuged and washed with hexane several times. The residue was dried over, and purification was achieved by column chromatography in silica gel (Hexane:EtOAc, 1:1 and 1:2).

# 5. Synthesis and Characterization of TEMPO Asymmetrical Zinc Phthalocyanine (TEMPO-Asym-ZnPc)

Asymmetrical TEMPO substituted zinc phthalocyanine (TEMPO-Asym-ZnPc) was synthesized as follows: To the suspension of TEMPO-Pht (1.0 equiv) and 4-*tert*-butylphthalonitrile (10 equiv) in n-hexanol, Zn(CH_3_COO)_2_ (2.75 equiv) and a few drops DBU was added. It was stirred for 5 h at 130 ^o^C. After that, the reaction mixture was poured into n-hexane, and the precipitate was centrifuged and washed with hexane several times. The residue was dried over, and purification was achieved by column chromatography in silica gel (Hexane:THF, 5:1).

**Figure S1.** FT-IR spectrum of TEMPO-Asym-ZnPc.

**Figure S2.** MALDI-TOF MS spectrum of TEMPO-Asym-ZnPc.

**Figure S3.** EPR spectrum of TEMPO-Asym-ZnPc in THF (0.01 mg/mL).





**Figure S4.** UV-vis spectrum of TEMPO-Asym-ZnPc in DMF (1 mg/mL).

# 6. Synthesis of Linear Polymer via I1 Initiator

Styrene was dissolved in toluene under nitrogen atmosphere, and BPO (**I1**) and TEMPO-Asym-ZnPc were added to the stirring solution. It was stirred for 24 h at 130 ^o^C. Then, THF was added, and it was precipitated in acidified methanol. The precipitate was centrifuged and washed with methanol many times. TEMPO-Asym-ZnPc (10 mg, 1.1x10^-5^ x 1 TEMPO unit = 1.1 x 10^-5^ mol, 1 equiv), BPO (3.87 mg, 1.6 x 10^-5^ mol, 1.5 equiv), styrene (0.22 mL, 1.92 x 10^-3^ mol, 192 equiv) and toluene (0.22 mL) were used. Yield: 168 mg (84%).

# 7. Synthesis of Linear Polymer via I2 Initiator

2,2,6,6-tetramethyl-1-(1-phenyl ethoxy)piperidine (**I2**) was synthesized according to a modified literature procedure.^[2]^ Styrene was dissolved in toluene under nitrogen atmosphere, and BPO and TEMPO-Asym-ZnPc were added to the stirring solution. It was stirred for 24 h at 130 ^o^C. Then, THF was added, and it was precipitated in acidified methanol. The precipitate was centrifuged and washed with methanol many times. TEMPO-Asym-ZnPc (10 mg, 1.1 x 10^-5^ x 1 TEMPO unit = 1.1 x 10^-5^ mol, 1 equiv), I2 (4.18 mg, 1.6 x 10^-5^ mol, 1.5 equiv), styrene (0.22 mL, 1.92 x 10^-3^ mol, 192 equiv) and toluene (0.22 mL) were used. Yield: 221 mg (41%).

# 8. Synthesis of Star-shaped Polymer via I1 Initiator

Monomer(s), TEMPO-Sym-ZnPc, and **I1** were dissolved in toluene and degassed via nitrogen bubbling in the Schlenk tube. The tube was sealed and placed in 130 °C oil bath. After given time, the reaction was taken out from the oil bath and cooled down by removing the rubber septa. Every reaction resulted in a highly viscous solution. The solution was diluted with THF and subsequently precipitated into cold methanol (two times). Greenish polymers were obtained and dried overnight under vacuum. The color of the polymer varied based on Pc concentrations from dark to light green.

**Star Polymer 1 (SP1):** TEMPO-Sym-ZnPc (5 mg, 3.97 x 10^-6^ x 4 TEMPO units = 1.58 x 10^-5^ mol, 1 equiv), I1 (5.76 mg, 2.38 x 10^-5^ mol, 1.5 equiv), Styrene (0.54 mL, 4.76 x 10^-3^ mol, 300 equiv) and Toluene (0.54 mL) were used. Yield: 336 mg (68%).

**Star Polymer 2 (SP2):** TEMPO-Sym-ZnPc (5 mg, 3.97 x 10^-6^ x 4 TEMPO units = 1.58 x 10^-5^ mol, 1 equiv), I1 (5.76 mg, 2.38 x 10^-5^ mol, 1.5 equiv), Styrene (0.18 mL, 1.58 x 10^-3^ mol, 100 equiv), VBCl (0.22 mL, 1.58 x 10^-3^ mol, 100 equiv) and Toluene (0.5 mL) were used. Yield: 218 mg (54%).

**Star Polymer 3 (SP3):** TEMPO-Sym-ZnPc (5 mg, 3.97 x 10^-6^ x 4 TEMPO units = 1.58 x 10^-5^ mol, 1 equiv), I1 (5.76 mg, 2.38 x 10^-5^ mol, 1.5 equiv), VBCl (0.67 mL, 1.58 x 10^-3^ mol, 100 equiv) and Toluene (0.67 mL) were used. Yield: 569 mg (79%).

#

# 9. Synthesis of Star-shaped Polymer via I2 Initiator

Monomer(s), TEMPO-Sym-ZnPc, and **I2** were dissolved in toluene and degassed via nitrogen bubbling in the Schlenk tube. The tube was sealed and placed in 130 °C oil bath. After given time, the reaction was taken out from the oil bath and cooled down by removing the rubber septa. Every reaction resulted in a highly viscous solution. The solution was diluted with THF and subsequently precipitated into cold methanol (two times). Greenish polymers were obtained and dried overnight under vacuum. The color of the polymer varied based on Pc concentrations from dark to light green.

**Star Polymer 4 (SP4):** TEMPO-Sym-ZnPc (5 mg, 3.97 x 10^-6^ x 4 TEMPO units = 1.58 x 10^-5^ mol, 1 equiv), I2 (16.52 mg, 6.32 x 10^-5^ mol, 4 equiv), Styrene (0.54 mL, 4.76 x 10^-3^ mol, 301 equiv) and Toluene (0.54 mL) were used. Yield: 364 mg (74%).

**Star Polymer 5 (SP5):** TEMPO-Sym-ZnPc (5 mg, 3.97 x 10^-6^ x 4 TEMPO units = 1.58 x 10^-5^ mol, 1 equiv), I2 (16.52 mg, 6.32 x 10^-5^ mol, 4 equiv), Styrene (0.18 mL, 1.58 x 10^-3^ mol, 100 equiv), VBCl (0.22 mL, 1.58 x 10^-3^ mol, 100 equiv) and Toluene (0.5 mL) were used. Yield: 460 mg (40%).

**Star Polymer 6 (SP6):** TEMPO-Sym-ZnPc (5 mg, 3.97 x 10^-6^ x 4 TEMPO units = 1.58 x 10^-5^ mol, 1 equiv), I2 (16.52 mg, 6.32 x 10^-5^ mol, 4 equiv), VBCl (0.67 mL, 1.58 x 10^-3^ mol, 100 equiv) and Toluene (0.67 mL) were used. Yield: 713 mg (98%).


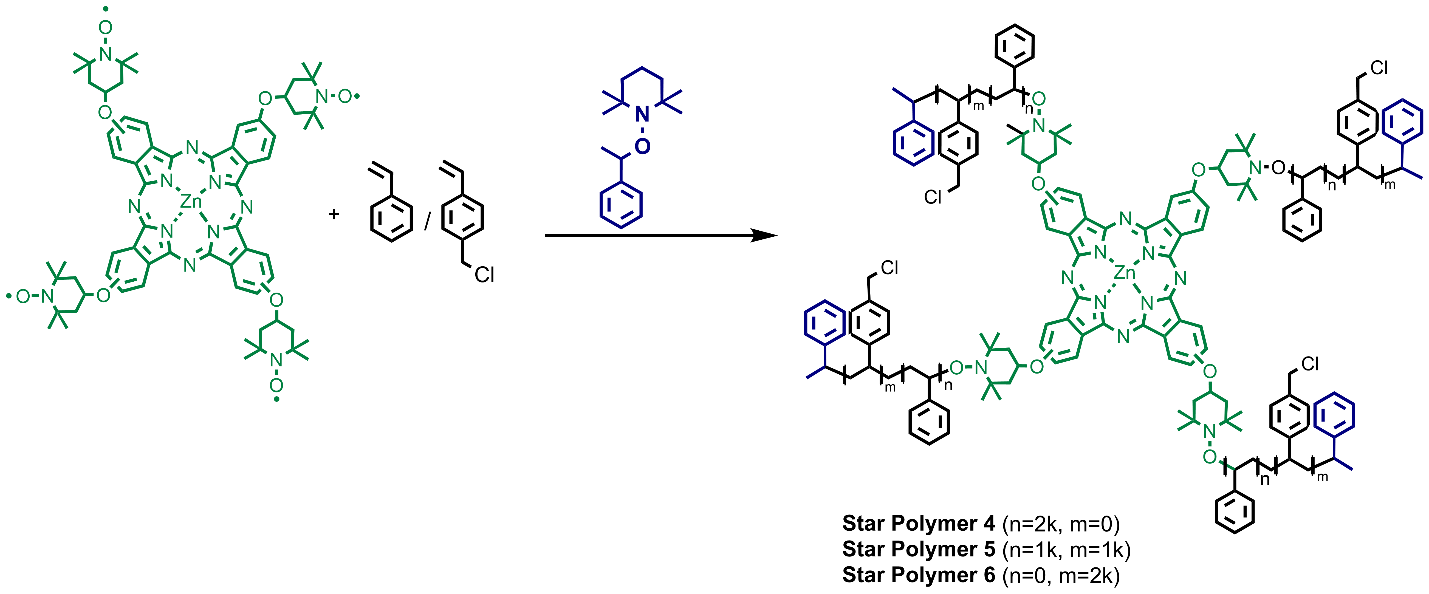


**Molecular Weight Calculation**

$$\int\left( \boldsymbol{CH} \right)\boldsymbol{\delta}\left( \boldsymbol{1}\boldsymbol{H} \right)\boldsymbol{=3.6}$$

$$\int\boldsymbol{(Ph-H)}\boldsymbol{\delta}\left( \boldsymbol{1}\boldsymbol{H} \right)\boldsymbol{=6.2-7.25}$$

$$nPS=\frac{a(Ph-H).m(CH).n(CH)}{a(CH). m(Ph-H)}$$

$$Mn=n.Mw\left( St \right)+Mw(initiator)$$

$a$ **=** the area or intensity of the ^1^H NMR peak of moiety**.**

$m$ = the number of protons of species.

$n$ **=** the number of repeating units of moiety**.**

**Molar Ratios and Mass percentages**

| **Polymers** | ***m/n* ratio^a^** | ***m/n* ratio^b^** | **Mass %^c^** | **Mass %^d^** |
| --- | --- | --- | --- | --- |
| **SP1** | - | - | 0 | 0 |
| **SP2** | - | - | 100 | 100 |
| **SP3** | 1 | 0.95 | 50 | 48 |
| **SP4** | - | - | 0 | 0 |
| **SP5** | - | - | 100 | 100 |
| **SP6** | 1 | 0.95 | 50 | 48 |

^a^Theoretical ratio of VBCl monomer. ^b^Calculated ratio of VBCl monomer by ^1^H-NMR. ^c^Theoretical mass percentage of VBCl monomer. ^d^Calculated mass percentage of VBCl monomer

# 10. Characterization of Polymers

#



**Figure S5.** FT-IR spectra of TEMPO-Asym-ZnPc (light green), **LP1** (red) and **LP2** (blue)





**Figure S6.** UV-vis spectra of **LP1** (red) and **LP2** (blue) in 1 mg/mL DMF.

#
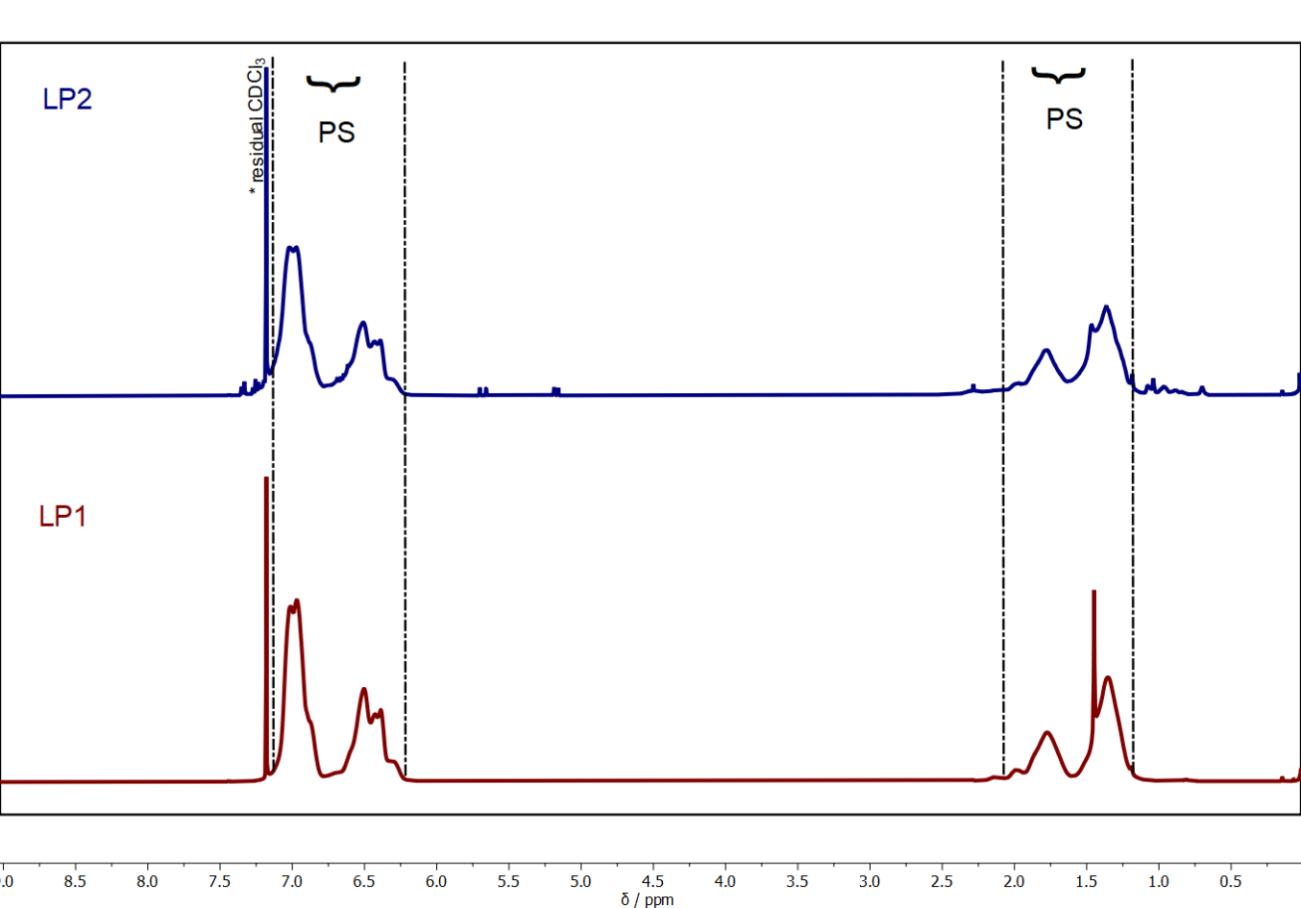


**Figure S7.** ^1^H-NMR spectra of **LP1** (red) and **LP2** (blue) in CDCl_3_.

#
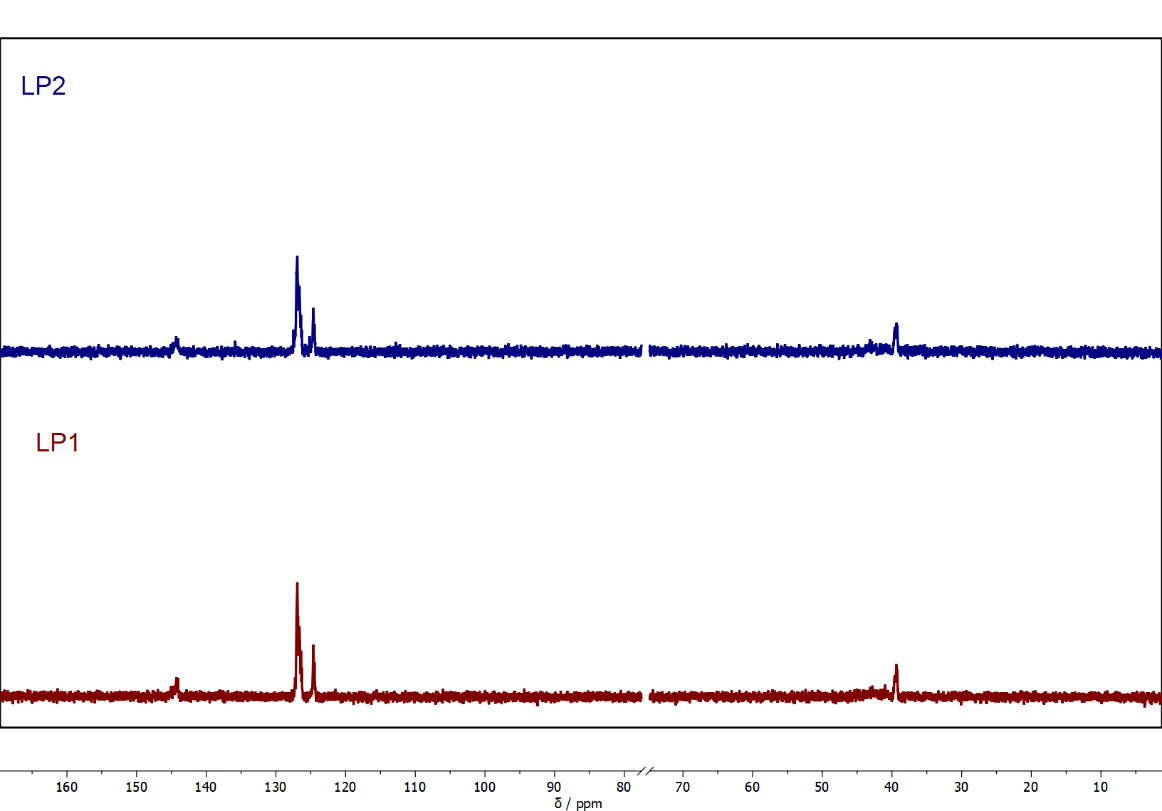


**Figure S8.** ^13^C-NMR spectra of **LP1** (red) and **LP2** (blue) in CDCl_3_.


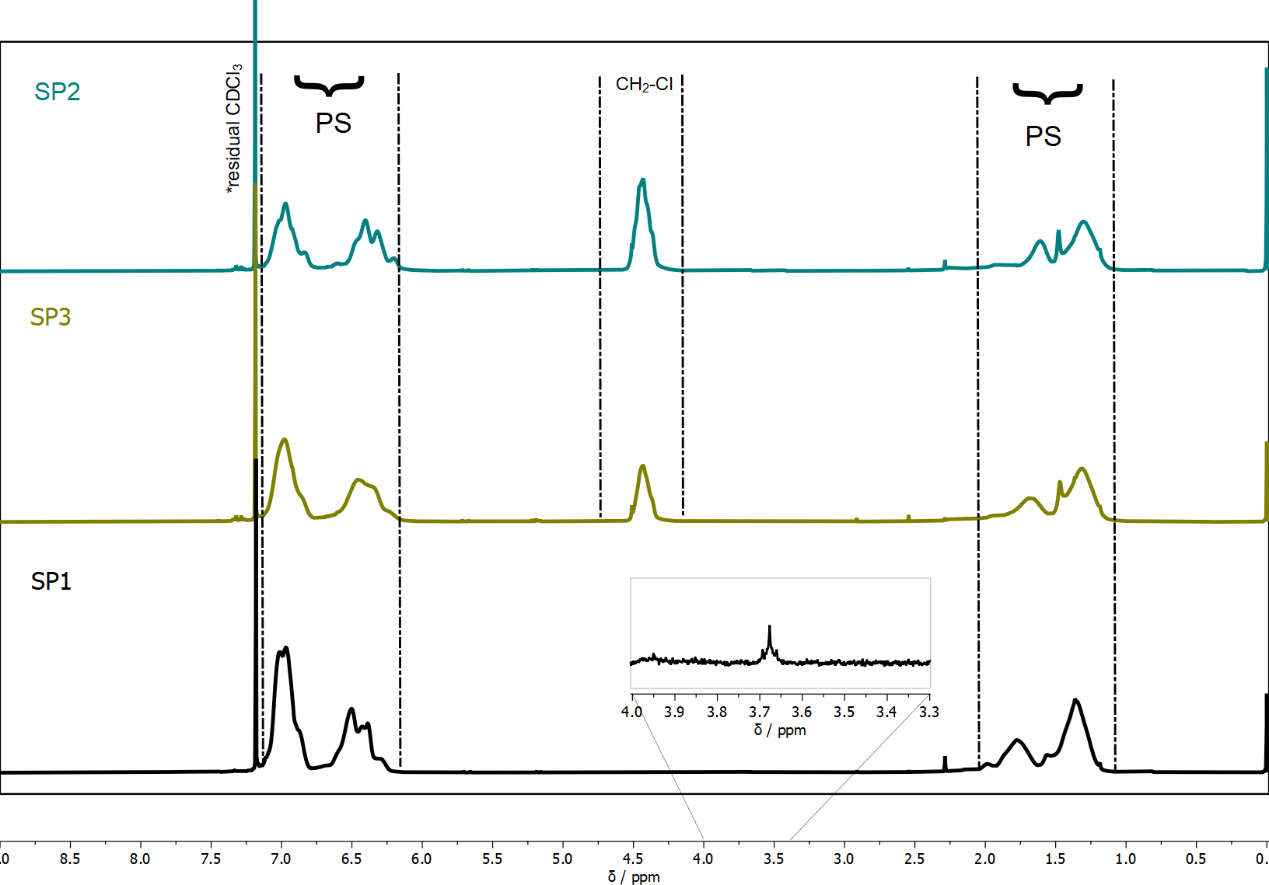


**Figure S9.** ^1^H-NMR spectra of **SP1** (black), **SP2** (yellow) and **SP3** (blue) in CDCl_3_.


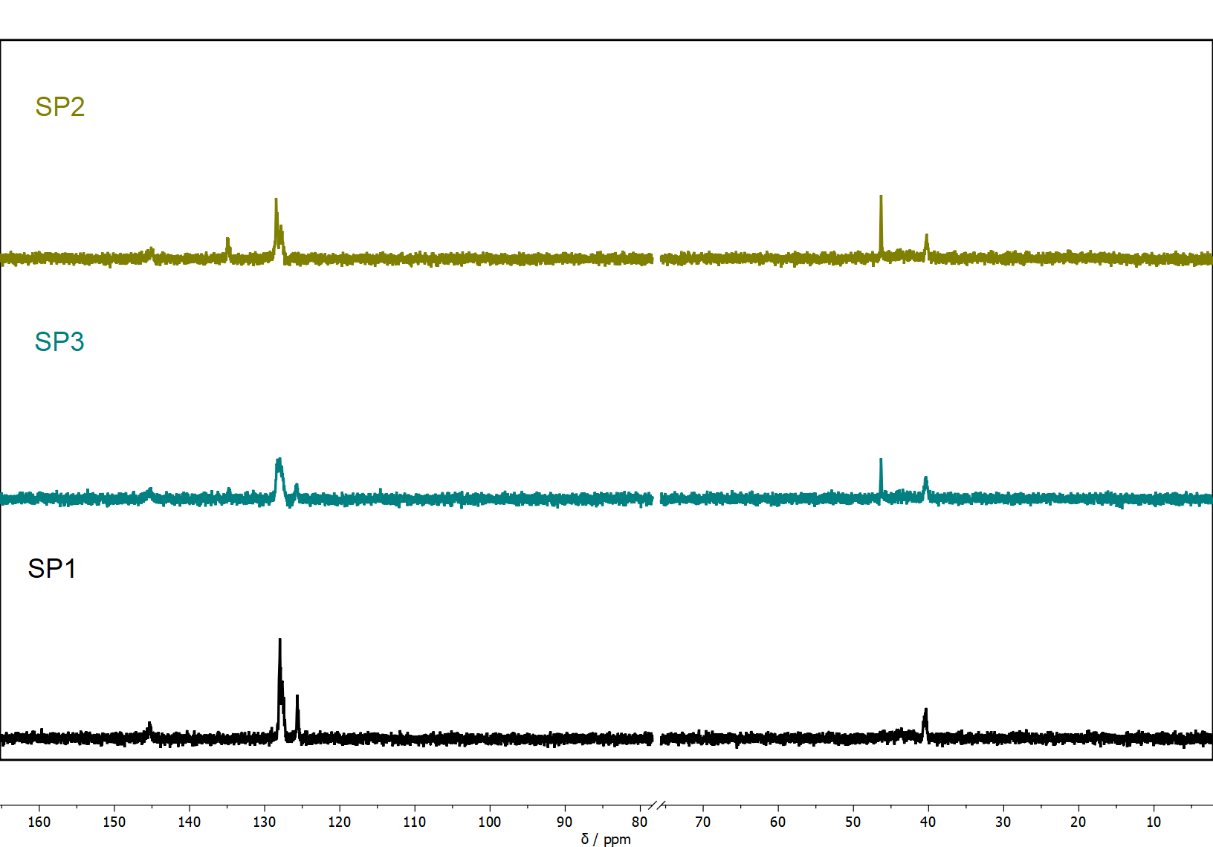


**Figure S10.** ^13^C-NMR spectra of **SP1** (black), **SP2** (yellow) and **SP3** (blue) in CDCl_3_.


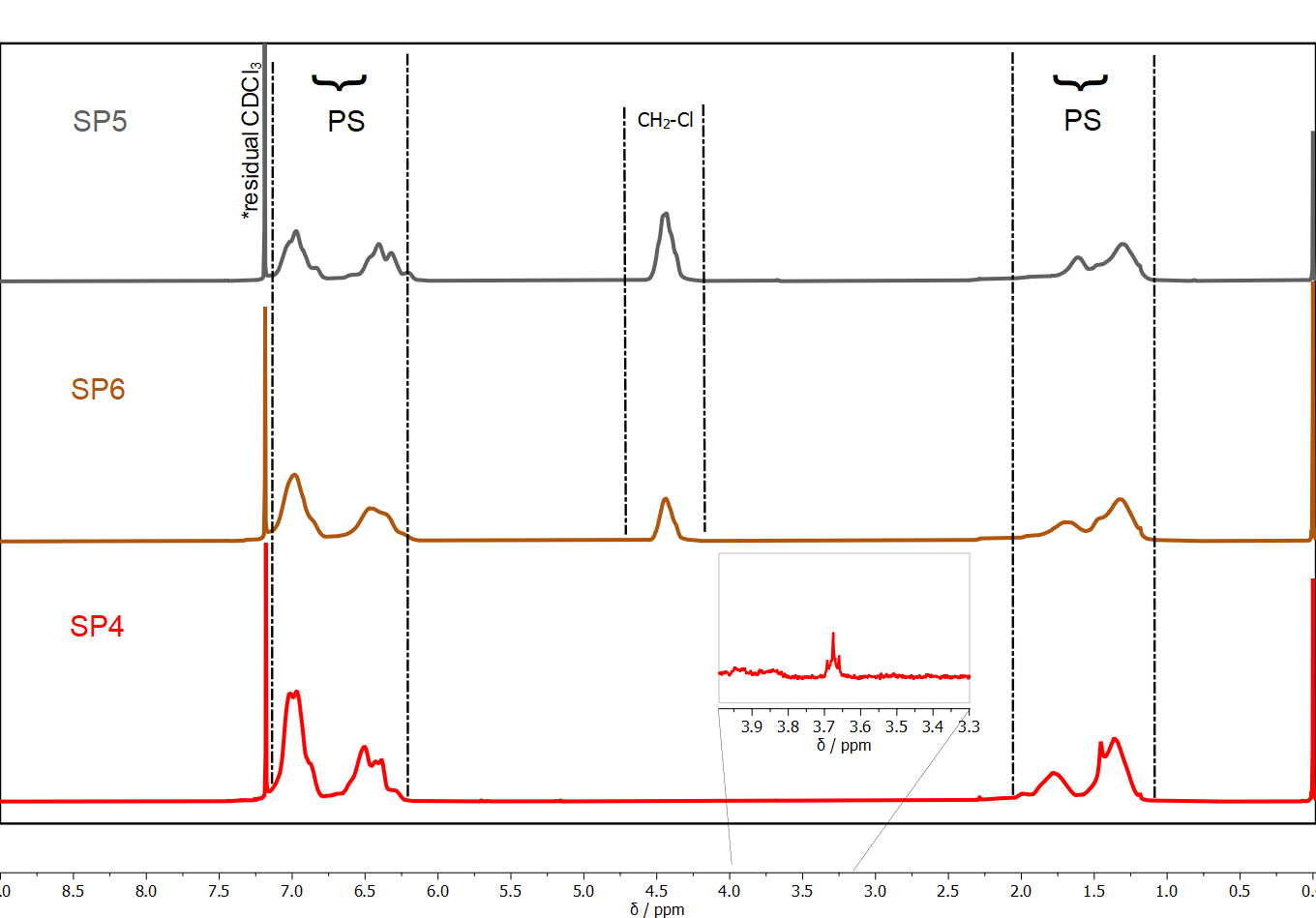


**Figure S11**. ^1^H-NMR spectra of **SP4** (red), **SP5** (gray) and **SP6** (orange) in CDCl_3_.


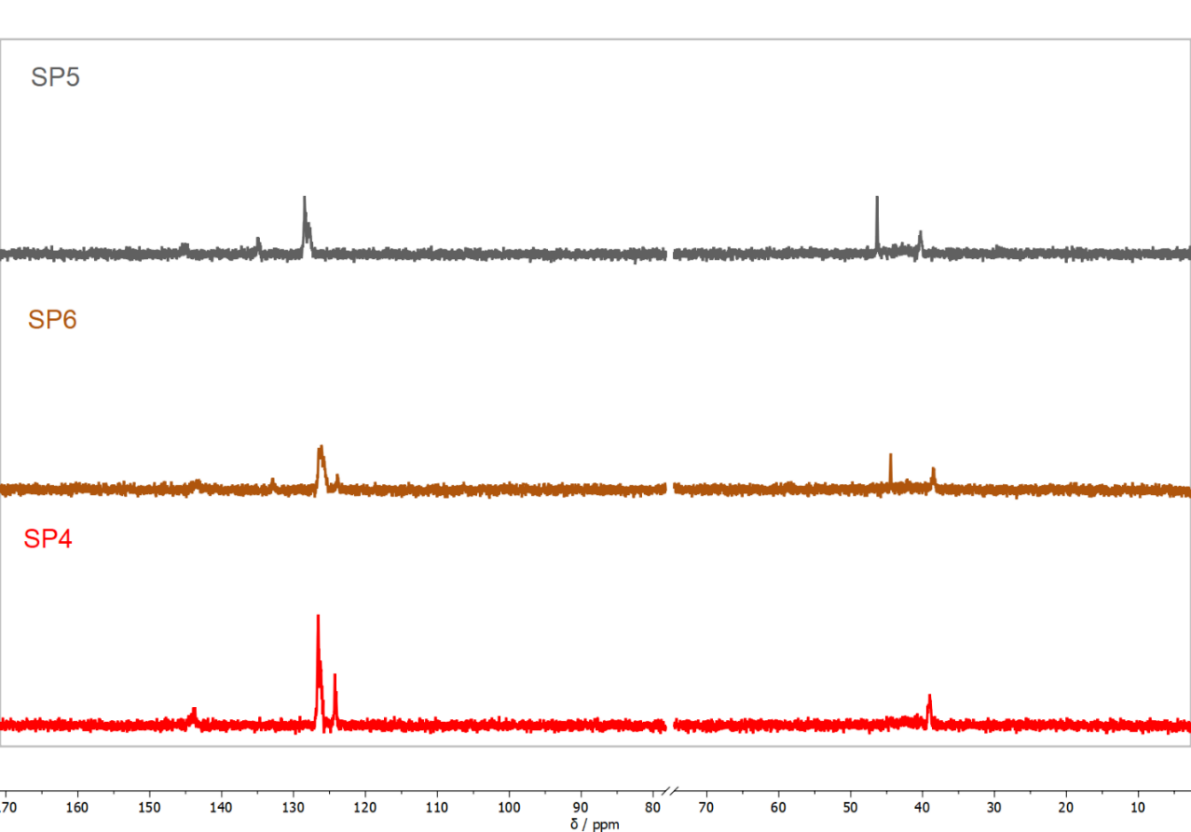


**Figure S12.** ^13^C-NMR spectra of **SP4** (red), **SP5** (gray) and **SP6** (orange) in CDCl_3_.





# Figure S13. EPR spectra of TEMPO-Sym-ZnPc (green), SP1 (black), SP2 (yellow), and SP3 (blue) in THF (0.01 g/mL).





# Figure S14. EPR spectra of TEMPO-Sym-ZnPc (green), SP4 (red), SP5 (gray), and SP6 (orange) in THF (0.01 g/mL).

#



# Figure S15. UV-vis spectra of star-shaped polymer SP1 (black), SP2 (yellow), and SP3 (blue) in DMF (1 mg/mL).





**Figure S16.** UV-vis spectra of star-shaped polymer **SP4** (red), **SP5** (gray), and **SP6** (orange) in DMF (1 mg/mL).

#



**Figure S17.** Fluorescence emission spectra of the **SP1** (black), **SP2** (yellow), and **SP3** (blue) in DMF (excitation wavelength = 612 nm).





**Figure S18.** Fluorescence emission spectra of the **SP4** (red), **SP5** (gray), and **SP6** (orange) in DMF (excitation wavelength = 612 nm).

**Figure S19.** FT-IR spectra of **SP1** (black), **SP2** (yellow) and **SP3** (blue).

**Figure S20.** FT-IR spectra of **SP4** (red), **SP5** (gray) and **SP6** (orange).

# References

[1] S. E. Korkut, D. Akyüz, K. Özdoğan, Y. Yerli, A. Koca, M. K. Şener, *Dalton Transactions* **2016**, *45*, 3086.

[2] M. Herder and Jean-Marie Lehn, *J. Am. Chem. Soc.* **2018**, 140, 7647.
